# Compressible turbulent plane channel DNS datasets

**DOI:** 10.1016/j.dib.2024.110737

**Published:** 2024-07-14

**Authors:** G.A. Gerolymos, I. Vallet

**Affiliations:** Sorbonne Université, Faculty of Science and Engineering, 4 place Jussieu, 75005 Paris, France

**Keywords:** Turbulence, DNS (direct numerical simulation), Compressible aerodynamics, Turbulent boundary-layer

## Abstract

The database contains detailed statistics of compressible turbulent plane channel (TPC) flow, obtained from direct numerical simulation (DNS), with a very-high-order massively parallel solver of the compressible Navier-Stokes equations. It contains datasets for 25 different flow conditions determined by the corresponding HCB friction Reynolds number and centerline Mach number, covering the ranges $100\;⪅\;Re_{\tau^{★}}\;⪅\;1000$ and $0.3\;⪅\;{\bar{M}}_{{\text{CL}}_{x}}\;⪅\;2.5$. All calculations are for strictly isothermal wall conditions at temperature $T_{w}\;=\;298\;\text{K}$ in a medium-size (MB) computational box (8πδ×2δ×4πδ where 2δ is the channel-height). Statistics (moments and pdfs) were collected after the elimination of the transient, and post-processed to create the dataset, which contains only plain text (.txt) space-separated multicolumn files for ease of use. The dataset for each flow-condition is tagged by the values of $\left(Re_{\tau^{★}},{\bar{M}}_{{\text{CL}}_{x}}\right)$ and is organized in 4 directories: (0) global data files, (1) profiles and budgets (meanflow profiles, velocity-moments up to 6-order, budgets of Reynolds-stresses transport, turbulent fluxes appearing in transport equations for velocity-moments and thermodynamic quantities, correlation coefficients between thermodynamic variables, and skewness and flatness profiles) as a function of the wall-distance, (2) single-variable probability density functions (pdfs) for numerous flow quantities at selected wall-normal distances, and (3) two-variable joint pdfs for numerous couples of flow-quantities at the same selected wall-normal distances.

Specifications Table

**Table:** 

Subject	Engineering, Aerospace Engineering
Specific subject area	Compressible turbulent boundary-layer, Direct numerical simulation (DNS)
Type of data	Raw, Analyzed
Data format	Plain text (.txt) files
Data collection	The data were obtained from DNS (direct numerical simulation) computations using a massively parallel very-high-order DNS solver [5] with air thermodynamics. For each of the 25 flow-conditions the flowfield was initialized using remeshing and rescaling (geometric and aerothermodynamic) from existing baseline simulations [5], [6], computations were run for a physical time $t_{\text{CNVRG}}$ to eliminate the transient, then continued activating the online acquisition of statistics (moments and pdfs, sampled at every iteration) for an observation time $t_{\text{OBS}}$. Fully automated post-processing was then applied to calculate Reynolds and Favre averages and fluctuation-moments of relevant quantities [6], [8], [9].
Data source location	Institution: Sorbonne Université
	Campus: Pierre-et-Marie-Curie, Faculty of Science and Engineering
	City: Paris
	Country: France [FRA]
	Computing centers: TGCC[19], CINES[2], IDRIS[11], JSC[13]
Data accessibility	Repository name: Mendeley Data
	Data identification number (doi): 10.17632/wt8t5kxzbs.1
	Direct URL to data: https://data.mendeley.com/datasets/wt8t5kxzbs/1
Related research articles	[8] G.A. Gerolymos and I. Vallet, Scaling of pressure fluctuations in compressible turbulent plane channel flow, *Journal of Fluid Mechanics***958** (2023) A19 doi:10.1017/jfm.2023.42
	[9] G.A. Gerolymos and I. Vallet, Total and static temperature statistics in compressible turbulent plane channel flow, *Journal of Fluid Mechanics***978** (2024) A25 doi:10.1017/jfm.2023.1034

## 1 Value of the Data

• A particular effort was made to construct a matrix (Cartesian grid) of $\left(Re_{\tau^{★}},{\bar{M}}_{{\text{CL}}_{x}}\right)$-values, allowing the investigation of $Re_{\tau^{★}}$-effects at constant ${\bar{M}}_{{\text{CL}}_{x}}$, and of ${\bar{M}}_{{\text{CL}}_{x}}$-effects at constant $Re_{\tau^{★}}$ [8], [9].
• The present datasets include a large number of turbulent correlations which are not available in existing datasets of compressible turbulent plane clannel (TPC) or turbulent boundary-layer (TBL) flows
  (i) profiles of the complete set of moments and correlations (orders 2 and 3) of turbulent fluctuations of thermodynamic variables [7]
  (ii) profiles of all velocity fluctuations moments and correlations (up to 6-order for Reynolds fluctuations and up to 4-order for Favre fluctuations)
  (iii) profiles of all fluxes for turbulent transport by the fluctuating velocities appearing in the transport equations of second moments of velocity and thermodynamic variables [6]
  (iv) single-variable and joint two-variable pdfs (probability density functions) of a large number of flow-quantities [8], [9]
  in addition to profiles of meanflow, Reynolds-stresses (and the detailed budgets of their transport equations) and turbulent heatfluxes.
• The database includes $Re_{\tau^{★}}\;≊1000$ datasets for which a common inner/outer region becomes clearly visible in the profiles.
• The datasets can be easily used as reference data for the analysis and modeling of compressible aerodynamic wall-flows.

## 2 Background

High-speed aerodynamics in aerospace applications require highly accurate DNS data, both to enhance our understanding of the complex multiscale aerothermodynamic phenomena [18], [21] observed in compressible wall-turbulence and to use as reference data for the development and validation of advanced turbulence models [15]. Data of compressible turbulent boundary-layers (TBL) [1], [20] and compressible turbulent plane channels (TPC) [14], [22] have largely contributed to this goal.

The principal parameters determining the flow physics of these canonical compressible turbulent wall-flows are the couple of Reynolds and Mach numbers and the wall-temperature (wall-heatflux) condition [22]. Notice that most of compressible wall-turbulence studies (including the present database) use a Sutherland’s law for the dynamic viscosity [6, p. 706], so that the wall-temperature (TPC) or the external flow temperature (TBL) in K is an additional parameter of flow conditions. In the case of canonical compressible TPC flow the wall-heatflux is determined by frictional heating [9], [17], so that the flow conditions are determined by the triplet $\left(Re_{\tau^{★}},{\bar{M}}_{{\text{CL}}_{x}},{\bar{T}}_{w}\right)$. Computations which control the wall-heatflux by an artificial sink-term in the energy equation [21] are not considered in the present database.

The motivation for the creation of the present database is twofold: (a) the development of a $\left(Re_{\tau^{★}},{\bar{M}}_{{\text{CL}}_{x}}\right)$-matrix of datasets whose need was felt in earlier work [6], [14], and (b) the accurate computation of a large number of correlations [7], [15] that are not systematically calculated or openly accessible.

In line with the description of the flow model used in the computations [6, p. 706], we will note $u_{i}\;\in \left\{u,v,w\right\}$ the velocity components along the Cartesian axes $x_{i}\in \;\left\{x,y,z\right\}$ (streamwise, wall-normal, spanwise), $V\;:=\;\surd (u^{2}+v^{2}+w^{2})$ the velocity modulus, ρ the density, T the temperature, p the pressure, h the enthalpy, $h_{t}:=h+\frac{1}{2}V^{2}$ the total enthalpy, s the entropy, a the sound speed, M:=V/a the Mach number with components $M_{i}\;:=\;u_{i}/a\;\in \;\left\{M_{x},M_{y},M_{z}\right\}$, μ the dynamic viscosity, $\mu_{b}$ the bulk viscosity, λ the heat conductivity, $c_{p}$ the specific heat capacity at constant pressure, $\gamma \;:=\;{\left(\partial \ln p/\partial \ln\rho \right)}_{s}$ the isentropic exponent, and $R_{g}$ the gas constant. We use the notations $\left(\cdot \right)\;=\;\bar{\left(\cdot \right)}\;+\;{\left(\cdot \right)}^{\prime }\;=\;\tilde{\left(\cdot \right)}\;+\;{\left(\cdot \right)}^{\prime \prime }$ for Reynolds and Favre averages and fluctuations. The equivalent notation $\left(\cdot \right)\;=\;\langle \left(\cdot \right)\rangle \;+\;{\left(\cdot \right)}^{\prime }\;=\;\left\{\left(\cdot \right)\right\}\;+\;{\left(\cdot \right)}^{\prime \prime }$ for Reynolds and Favre averages is used in the comments included in the datafiles. We use 3 different systems of inner-units in the datafiles for the nondimensionalization of the data: (a) ${\left(\cdot \right)}^{+}\text{-units:}\;\left\{{\bar{\tau }}_{w},{\bar{\mu }}_{w},{\bar{\rho }}_{w}\right\}$, (b) ${\left(\cdot \right)}^{★}\text{-units:}\;\left\{{\bar{\tau }}_{w},\bar{\mu }\left(y\right),\bar{\rho }\left(y\right)\right\}$, and (c) ${\left(\cdot \right)}^{\#}\text{-units:}\;\left\{{\bar{\tau }}_{w},{\bar{\mu }}_{\text{CL}},{\bar{\rho }}_{\text{CL}}\right\}$, where ${\bar{\tau }}_{w}$ is the wall shear-stress. When appropriate, outer-scaling $\left\{{\bar{u}}_{\text{CL}},\delta ,{\bar{\rho }}_{\text{CL}}\right\}$ is also used.

We define

(1a) $$\begin{array}{c} {\bar{M}}_{{\text{CL}}_{x}}:=\bar{\left(\frac{u_{\text{CL}}}{a_{\text{CL}}}\right)}\;;\;Re_{\tau^{★}}:=\frac{\sqrt{{\bar{\tau }}_{w}{\bar{\rho }}_{\text{CL}}}\delta }{{\bar{\mu }}_{\text{CL}}}=\delta^{★}=\delta^{\#} \end{array}$$

(1b) $$\begin{array}{c} y^{★}:=\frac{\sqrt{{\bar{\tau }}_{w}\bar{\rho }\left(y\right)}\;y}{\bar{\mu }\left(y\right)}\;;\;y^{\#}:=\frac{\sqrt{{\bar{\tau }}_{w}{\bar{\rho }}_{\text{CL}}}\;y}{{\bar{\mu }}_{\text{CL}}}\;;\;y^{+}:=\frac{\sqrt{{\bar{\tau }}_{w}{\bar{\rho }}_{w}}\;y}{{\bar{\mu }}_{w}} \end{array}$$

the centerline streamwise Mach number, the HCB friction Reynolds number and the nondimensional wall-distances in the 3 inner-units systems.

## 3 Data Description

**Fig. 1:**
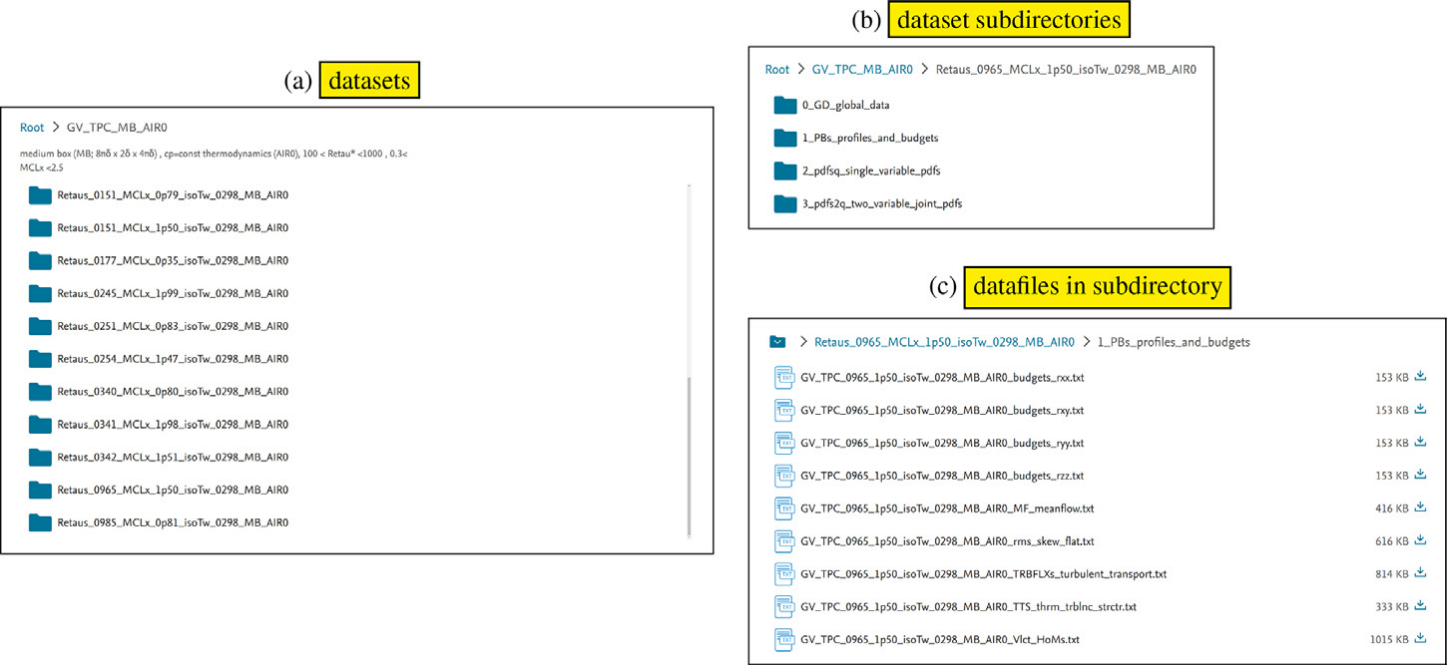
View of (a) the dataset directories for each $\left(Re_{\tau^{★}},{\bar{M}}_{{\text{CL}}_{x}},{\bar{T}}_{w}\right)$ contained in the database directory GV_TPC_MB_AIR0, (b) the subdirectories contained in each dataset, and (c) the plain-text (.txt) files contained in each of these subdirectories (the typical dataset Retaus_0965_MCLx_1p50_isoTw_0298_MB_AIR0 will be used to describe the datasets).

The database is contained in directory GV_TPC_MB_AIR0, where AIR0 denotes the flow model detailed in [6, p. 706], MB denotes the computational-box-size (8πδ×2δ×4πδ), TPC is the acronym for turbulent plane channel, and the prefix GV was used to identify the present database. The parent directory GV_TPC_MB_AIR0 contains 25 subdirectories Fig. 1a, each corresponding to a different flow-condition (Table 1), *eg* Retaus_0965_MCLx_1p50_isoTw_0298_MB_AIR0 corresponds to $\left(Re_{\tau^{★}},{\bar{M}}_{{\text{CL}}_{x}}\right)\;=\;\left(965,1.50\right)$ with strictly isothermal wall-condition at $T_{w}=298\;K$. The actual data are plain-text (.txt) files which are organized in 4 subdirectories Fig. 1b

• 0_GD_global_data (§3.1)
• 1_PBs_profiles_and_budgets (§3.2)
• 2_pdfsq_single_variable_pdfs (§3.3)
• 3_pdfs2q_two_variable_joint_pdfs (§3.4)

**Table 1:** Sampling parameters for averages, pdfs and joint pdfs [$Re_{\tau^{★}}$ is the friction Reynolds number in HCB-scaling (1); ${\bar{M}}_{{\text{CL}}_{x}}$ is the centerline Mach number (1); Δt is the computational time-step [8]; $\Delta t_{s}$, $\Delta t_{s_{q}}$, $\Delta t_{s_{2q}}$ are the sampling time-steps for averages, pdfs, and joint pdfs; $t_{\text{OBS}}$, $t_{{\text{OBS}}_{q}}$, $t_{{\text{OBS}}_{2q}}$ are the sampling observation intervals for averages, pdfs and joint pdfs; $N_{{\mathrm{bins}}_{q}}$, $N_{{\mathrm{bins}}_{2q}}$ are the sampling bins for the calculation of pdfs and joint pdfs; $N_{s}$, $N_{s_{q}}$, $N_{s_{2q}}$ is the number of samples involved in the calculation of pdfs and joint pdfs; ${\left(\cdot \right)}^{+}$ denotes wall-units]

			PBs; $\Delta t_{s}\;\;=\;\;\Delta t$		pdfs; $\Delta t_{s_{q}}\;\;=\;\;\Delta t$			joint pdfs; $\Delta t_{s_{2q}}\;\;=\;\;\Delta t$		
$Re_{\tau^{★}}$	${\bar{M}}_{{\text{CL}}_{x}}$	$t_{\text{OBS}}^{+}$	$N_{s}\;\;\;\;\;$	$t_{{\text{OBS}}_{q}}^{+}$	$N_{{\mathrm{bins}}_{q}}$	$N_{s_{q}}\;\;\;\;$	$t_{{\text{OBS}}_{2q}}^{+}$	$N_{{\mathrm{bins}}_{2q}}\;\;\;$	$N_{s_{2q}}\;\;\;\;$	
102.7	1.506	7392	$613\;\times \;\;10^{9}$	5894	200	$489\;\times \;\;10^{9}$	3249	200×200	$269\;\times \;\;10^{9}$	
100.4	1.819	7648	$398\;\times \;\;10^{9}$	6239	200	$325\;\times \;\;10^{9}$	3418	200×200	$178\;\times \;\;10^{9}$	
100.4	1.918	9296	$419\;\times \;\;10^{9}$	8092	200	$365\;\times \;\;10^{9}$	4717	200×200	$213\;\times \;\;10^{9}$	
98.9	2.015	6557	$264\;\times \;\;10^{9}$	5519	200	$222\;\times \;\;10^{9}$	3483	200×200	$140\;\times \;\;10^{9}$	
97.3	2.110	9589	$341\;\times \;\;10^{9}$	7832	200	$279\;\times \;\;10^{9}$	3367	200×200	$120\;\times \;\;10^{9}$	
98.5	2.218	8108	$235\;\times \;\;10^{9}$	6270	200	$182\;\times \;\;10^{9}$	3711	200×200	$107\;\times \;\;10^{9}$	
104.8	0.324	7786	$295\;\times \;\;10^{9}$	6532	200	$248\;\times \;\;10^{9}$	3236	200×200	$123\;\times \;\;10^{9}$	
106.0	0.793	9097	$129\;\times \;\;10^{9}$	6829	200	$97\;\times \;\;10^{9}$	4081	200×200	$58\;\times \;\;10^{9}$	
111.7	2.020	6327	$362\;\times \;\;10^{9}$	5099	200	$291\;\times \;\;10^{9}$	3890	200×200	$222\;\times \;\;10^{9}$	
112.7	2.488	6629	$2880\;\times \;\;10^{9}$	5168	1000	$2245\;\times \;\;10^{9}$	5168	200×200	$2245\;\times \;\;10^{9}$	
113.6	1.507	10496	$136\;\times \;\;10^{9}$	8015	200	$104\;\times \;\;10^{9}$	4996	200×200	$65\;\times \;\;10^{9}$	
134.2	1.991	7602	$539\;\times \;\;10^{9}$	4729	1000	$336\;\times \;\;10^{9}$	3419	200×200	$243\;\times \;\;10^{9}$	
143.1	0.324	7790	$304\;\times \;\;10^{9}$	6369	200	$249\;\times \;\;10^{9}$	3341	200×200	$131\;\times \;\;10^{9}$	
151.4	0.792	7060	$407\;\times \;\;10^{9}$	5693	200	$328\;\times \;\;10^{9}$	3211	200×200	$185\;\times \;\;10^{9}$	
151.5	1.503	9565	$209\;\times \;\;10^{9}$	7410	200	$162\;\times \;\;10^{9}$	4287	200×200	$94\;\times \;\;10^{9}$	
168.7	0.793	7673	$399\;\times \;\;10^{9}$	3494	1000	$182\;\times \;\;10^{9}$	3494	200×200	$182\;\times \;\;10^{9}$	
176.6	0.347	9695	$291\;\times \;\;10^{9}$	3939	1000	$118\;\times \;\;10^{9}$	3939	200×200	$118\;\times \;\;10^{9}$	
251.1	0.828	5902	$777\;\times \;\;10^{9}$	4670	200	$615\;\times \;\;10^{9}$	3642	200×200	$479\;\times \;\;10^{9}$	
253.9	1.471	6121	$519\;\times \;\;10^{9}$	4722	200	$400\;\times \;\;10^{9}$	3464	200×200	$294\;\times \;\;10^{9}$	
245.3	1.988	7373	$2689\;\times \;\;10^{9}$	6187	200	$2256\;\times \;\;10^{9}$	3774	200×200	$1376\;\times \;\;10^{9}$	
341.0	1.978	4973	$3165\;\times \;\;10^{9}$	3854	1000	$2453\;\times \;\;10^{9}$	3854	200×200	$2453\;\times \;\;10^{9}$	
341.8	1.513	6558	$1160\;\times \;\;10^{9}$	5383	200	$952\;\times \;\;10^{9}$	4048	200×200	$716\;\times \;\;10^{9}$	
339.6	0.798	7285	$682\;\times \;\;10^{9}$	5766	200	$540\;\times \;\;10^{9}$	4483	200×200	$420\;\times \;\;10^{9}$	
984.5	0.806	5547	$7077\;\times \;\;10^{9}$	4013	1000	$5120\;\times \;\;10^{9}$	4013	100×100	$5120\;\times \;\;10^{9}$	
965.2	1.505	7498	$12208\;\times \;\;10^{9}$	4303	1000	$7006\;\times \;\;10^{9}$	4303	100×100	$7006\;\times \;\;10^{9}$	

**Fig. 2:**
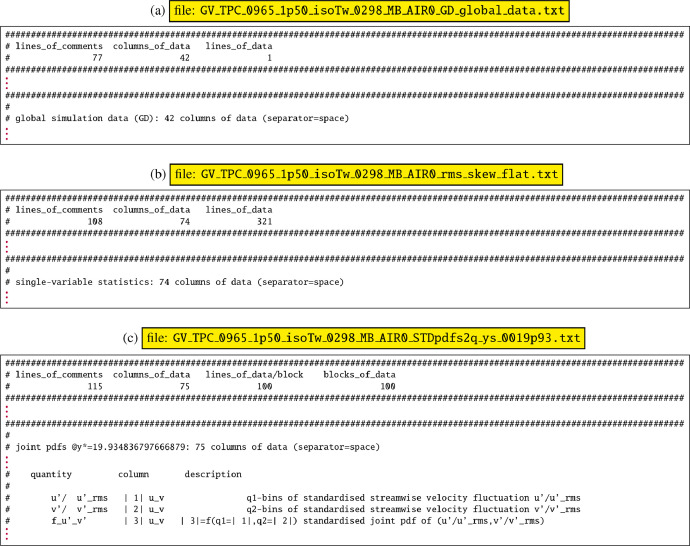
Typical datafile headers (a) directory 0_GD_global_data global datafiles containing 1 line of data, (b) directories 1_PBs_profiles_and_budgets and 2_pdfsq_single_variable_pdfs datafiles containing several lines of data, and (c) directory 3_pdfs2q_two_variable_joint_pdfs datafiles containing several blocks of data with each block containing several lines of data (line 3 of each datafile indicates the number of lines of comments before the numerical data, the number of space-separated columns in each dataline, and the lines of data or the lines of data per block and number of blocks).

Each individual datafile is tagged with the flow-conditions, to avoid mistakes (we will use the typical subdirectory Retaus_0965_MCLx_1p50_isoTw_0298_MB_AIR0 to describe the datasets). Each datafile contains a header (lines of comments starting with #), followed by the actual numerical data in space-separated columns. The comments are quite detailed and describe the data contained in the file. The numbers of comment-lines, of data-columns and of data-lines are systematically given in line 3 of each file (Fig. 2), as this can be helpful in reading the data by a script or program. All datafiles can be directly used for plotting [12].

### 3.1 GD (global data)

The subdirectory 0_GD_global_data of each dataset contains 3 files of global data.

• GV_TPC_0965_1p50_isoTw_0298_MB_AIR0_GD_global_data.txt tabulates global flow parameters: Reynolds and Mach numbers, integral thicknesses, nondimensional wall-to-centerline ratios or differences of averaged thermodynamic variables (p, ρ, T, h, s), *eg* [9, Tab. 1, p. A25-5]
• GV_TPC_0965_1p50_isoTw_0298_MB_AIR0_RB_box_and_resolution_data.txttabulates grid parameters and computational resolution data, *eg* [8, Tab. 1, p. A19-8]
• GV_TPC_0965_1p50_isoTw_0298_MB_AIR0_SI_dimensional_data.txt tabulates global flow parameters and box dimensions in SI units

These files contain the corresponding global data in human-readable format in the comments section (Fig. 2a), and are followed by a single line of space-separated data, which can be practical for extracting and assigning value(s), using awk [16], in scripts or plotting commands.

### 3.2 PBs (profiles and budgets)

All the datafiles in subdirectory 1_PBs_profiles_and_budgets of each dataset contain profiles as a function of the wall-distance. Therefore, the first 4 columns in each file tabulate wall-distance in different scalings (y/δ, $y^{★}$, $y^{\#}$, $y^{+}$). The following datafiles are available in subdirectory 1_PBs_profiles_and_budgets

• GV_TPC_0965_1p50_isoTw_0298_MB_AIR0_budgets_rxx.txttabulates the profiles of the terms in the transport-equations for the streamwise velocity variance $r_{xx}\;:=\bar{\rho u^{\prime \prime }u^{\prime \prime }}$
• GV_TPC_0965_1p50_isoTw_0298_MB_AIR0_budgets_rxy.txttabulates the profiles of the terms in the transport-equations for the velocity covariance $r_{xy}\;:=\bar{\rho u^{\prime \prime }v^{\prime \prime }}$
• GV_TPC_0965_1p50_isoTw_0298_MB_AIR0_budgets_ryy.txttabulates the profiles of the terms in the transport-equations for the wall-normal velocity variance $r_{yy}\;:=\bar{\rho v^{\prime \prime }v^{\prime \prime }}$
• GV_TPC_0965_1p50_isoTw_0298_MB_AIR0_budgets_rzz.txttabulates the profiles of the terms in the transport-equations for the spanwise velocity variance $r_{zz}\;:=\bar{\rho w^{\prime \prime }w^{\prime \prime }}$
• GV_TPC_0965_1p50_isoTw_0298_MB_AIR0_MF_meanflow.txttabulates profiles of meanflow-variables (dynamic and thermodynamic), both Reynolds- and when appropriate Favre-averaged, with different scalings
• GV_TPC_0965_1p50_isoTw_0298_MB_AIR0_rms_skew_flat.txttabulates the profiles of coefficients-of-variation ${\text{CV}}_{q^{\prime }}\;:=\;q_{\mathrm{rms}}^{\prime }/\bar{q}$ and higher-order moments (up to 6-order, skewness $S_{q^{\prime 3}}:=\bar{q^{\prime 3}}/q_{\mathrm{rms}}^{\prime 3}$, flatness $F_{q^{\prime 4}}:=\bar{q^{\prime 4}}/q_{\mathrm{rms}}^{\prime 4}$, superskewness $sS_{q^{\prime 5}}:=\bar{q^{\prime 5}}/q_{\mathrm{rms}}^{\prime 5}$ and superflatness $sF_{q^{\prime 6}}:=\bar{q^{\prime 6}}/q_{\mathrm{rms}}^{\prime 6}$ [4]) for all flow-variables (V, u, v, w, p, ρ, T, h, s, $h_{t}$) and Mach-numbers (M, $M_{x}$, $M_{y}$, $M_{z}$); contrary to all of the other files in subdirectory 1_PBs_profiles_and_budgets, statistics in this file were obtained from single-variable standardized pdfs (§3.3)
• GV_TPC_0965_1p50_isoTw_0298_MB_AIR0_TRBFLXs_turbulent_transport.txt tabulates fluxes which appear in different transport equations [6] ($\bar{u_{i}^{\prime \prime }}$, $\bar{\rho u_{i}^{\prime \prime }u_{j}^{\prime \prime }}$, $\bar{\rho u_{i}^{\prime \prime }u_{j}^{\prime \prime }u_{k}^{\prime \prime }}$, $\bar{\rho u_{i}^{\prime \prime }u_{j}^{\prime \prime }u_{k}^{\prime \prime }u_{\ell }^{\prime \prime }}$, $\bar{\rho^{\prime }u_{i}^{\prime }}$, $\bar{\rho^{\prime 2}u_{i}^{\prime }}$, $\bar{u_{i}^{\prime }u_{j}^{\prime }}$, $\bar{\rho^{\prime }u_{i}^{\prime }u_{j}^{\prime }}$, $\bar{p^{\prime }u_{i}^{\prime }}$, $\bar{p^{\prime 2}u_{i}^{\prime }}$, $\bar{p^{\prime }u_{i}^{\prime }u_{j}^{\prime }}$, $\bar{\rho h^{\prime \prime }u_{i}^{\prime \prime }}$, $\bar{\rho h^{\prime \prime 2}u_{i}^{\prime \prime }}$, $\bar{\rho h^{\prime \prime }u_{i}^{\prime \prime }u_{j}^{\prime \prime }}$, $\bar{\rho s^{\prime \prime }u_{i}^{\prime \prime }}$, $\bar{\rho s^{\prime \prime 2}u_{i}^{\prime \prime }}$, $\bar{\rho s^{\prime \prime }u_{i}^{\prime \prime }u_{j}^{\prime \prime }}$)
• GV_TPC_0965_1p50_isoTw_0298_MB_AIR0_TTS_thrm_trblnc_strctr.txt tabulates the coefficients-of-variation (CVs) and correlation coefficients up to 3-order between thermodynamic variables (p, ρ, T, h, s, a); also CVs and skewness of inverses (1/ρ, 1/T, 1/p, 1/a) and selected correlations including inverses
• GV_TPC_0965_1p50_isoTw_0298_MB_AIR0_Vlct_HoMs.txt tabulates all the moments of velocity-components in ${\left(\cdot \right)}^{★}\text{-units}$, up to 6-order for Reynolds fluctuations (${\bar{u}}_{i}$, $\bar{u_{i}^{\prime }u_{j}^{\prime }}$, $\bar{u_{i}^{\prime }u_{j}^{\prime }u_{k}^{\prime }}$, $\bar{u_{i}^{\prime }u_{j}^{\prime }u_{k}^{\prime }u_{\ell }^{\prime }}$, $\bar{u_{i}^{\prime }u_{j}^{\prime }u_{k}^{\prime }u_{\ell }^{\prime }u_{m}^{\prime }}$, $\bar{u_{i}^{\prime }u_{j}^{\prime }u_{k}^{\prime }u_{\ell }^{\prime }u_{m}^{\prime }u_{n}^{\prime }}$), and up to 4-order for Favre fluctuations (${\tilde{u}}_{i}$, $\left\{u_{i}^{\prime \prime }u_{j}^{\prime \prime }\right\}$, $\left\{u_{i}^{\prime \prime }u_{j}^{\prime \prime }u_{k}^{\prime \prime }\right\}$, $\left\{u_{i}^{\prime \prime }u_{j}^{\prime \prime }u_{k}^{\prime \prime }u_{\ell }^{\prime \prime }\right\}$)

Notice that with the definitions used for the budgets, the Reynolds-stresses are $-r_{ij}$. Statistics for correlations and budgets of many other quantities are available, and will be included in a future V2 version of the database.

### 3.3 pdfsq (single variable pdfs)

Probability density functions (pdfs) were acquired at every wall-normal station of the computational halfchannel grid. In subdirectory 2_pdfsq_single_variable_pdfs of each dataset we include pdf-data at selected wall-normal stations

• GV_TPC_0965_1p50_isoTw_0298_MB_AIR0_STDpdfsq_ys_0001p09.txt at $y^{★}\;=\;1.09$ (closest to $y^{★}\;=\;1$)
• GV_TPC_0965_1p50_isoTw_0298_MB_AIR0_STDpdfsq_ys_0002p02.txt at $y^{★}\;=\;2.02$ (closest to $y^{★}\;=\;2$)
• GV_TPC_0965_1p50_isoTw_0298_MB_AIR0_STDpdfsq_ys_0004p94.txt at $y^{★}\;=\;4.94$ (closest to $y^{★}\;=\;5$)
• GV_TPC_0965_1p50_isoTw_0298_MB_AIR0_STDpdfsq_ys_0010p14.txt at $y^{★}\;=\;10.14$ (closest to $y^{★}\;=\;10$)
• GV_TPC_0965_1p50_isoTw_0298_MB_AIR0_STDpdfsq_ys_0014p93.txt at $y^{★}\;=\;14.93$ (closest to $y^{★}\;=\;15$)
• GV_TPC_0965_1p50_isoTw_0298_MB_AIR0_STDpdfsq_ys_00..p...txt at the grid-stations closest to $y^{★}\;\in \left\{20,30,40,\ldots ,90\right\}$
• GV_TPC_0965_1p50_isoTw_0298_MB_AIR0_STDpdfsq_ys_0099p34.txt at $y^{★}\;=\;99.34$ (closest to $y^{★}\;=\;100$)
• GV_TPC_0965_1p50_isoTw_0298_MB_AIR0_STDpdfsq_ys_0...p...txt at the grid-stations closest to $y^{★}\;\in \left\{200,300,400,\ldots ,900\right\}$
• GV_TPC_0965_1p50_isoTw_0298_MB_AIR0_STDpdfsq_ys_0965p15.txt at centerline

Each datafile contains standardized pdfs for all flow-variables (V, u, v, w, p, ρ, T, h, s, $h_{t}$) and Mach-numbers (M, $M_{x}$, $M_{y}$, $M_{z}$). The number of pdf-bins is the same at each wall-normal station but the range $\left[q_{\min},q_{\max}\right]$ of sampling (bin width Δq) was adjusted for each variable and wall-normal station to include extreme events (§4.2).

### 3.4 pdfs2q (two-variable joint pdfs)

Joint 2-variable pdfs were also acquired at every wall-normal station of the computational halfchannel grid. In subdirectory 3_pdfs2q_two_variable_joint_pdfs of the datasets we include joint-pdf-data at the same selected wall normal stations as for the single-variable pdfs (§3.3).

• GV_TPC_0965_1p50_isoTw_0298_MB_AIR0_STDpdfs2q_ys_0001p09.txt.zip
• ⋯
• GV_TPC_0965_1p50_isoTw_0298_MB_AIR0_STDpdfs2q_ys_0965p15.txt.zip

Each datafile contains standardized joint pdfs at the corresponding wall-normal station for the 25 pairs (u,v), (v,w), (w,u), (ρ,u), (ρ,v), (ρ,w), (p,u), (p,v), (p,w), (h,u), (h,v), (h,w), (s,u), (s,v), (s,w), (p,ρ), (p,T), (p,s), (ρ,T), (ρ,s), (h,s), $\left(h,h_{t}\right)$, $\left(u,h_{t}\right)$, $\left(v,h_{t}\right)$, $\left(w,h_{t}\right)$. These are large files as they include data in 2-D variable-spaces for each pair, and were zipped using zip -r filename.zip filname (size reduced by a factor 4). The -r option is unnecessary and will be removed in a future V2 version of the database, to simplify usage.

## 4 Experimental Design, Materials and Methods

**Fig. 3:**
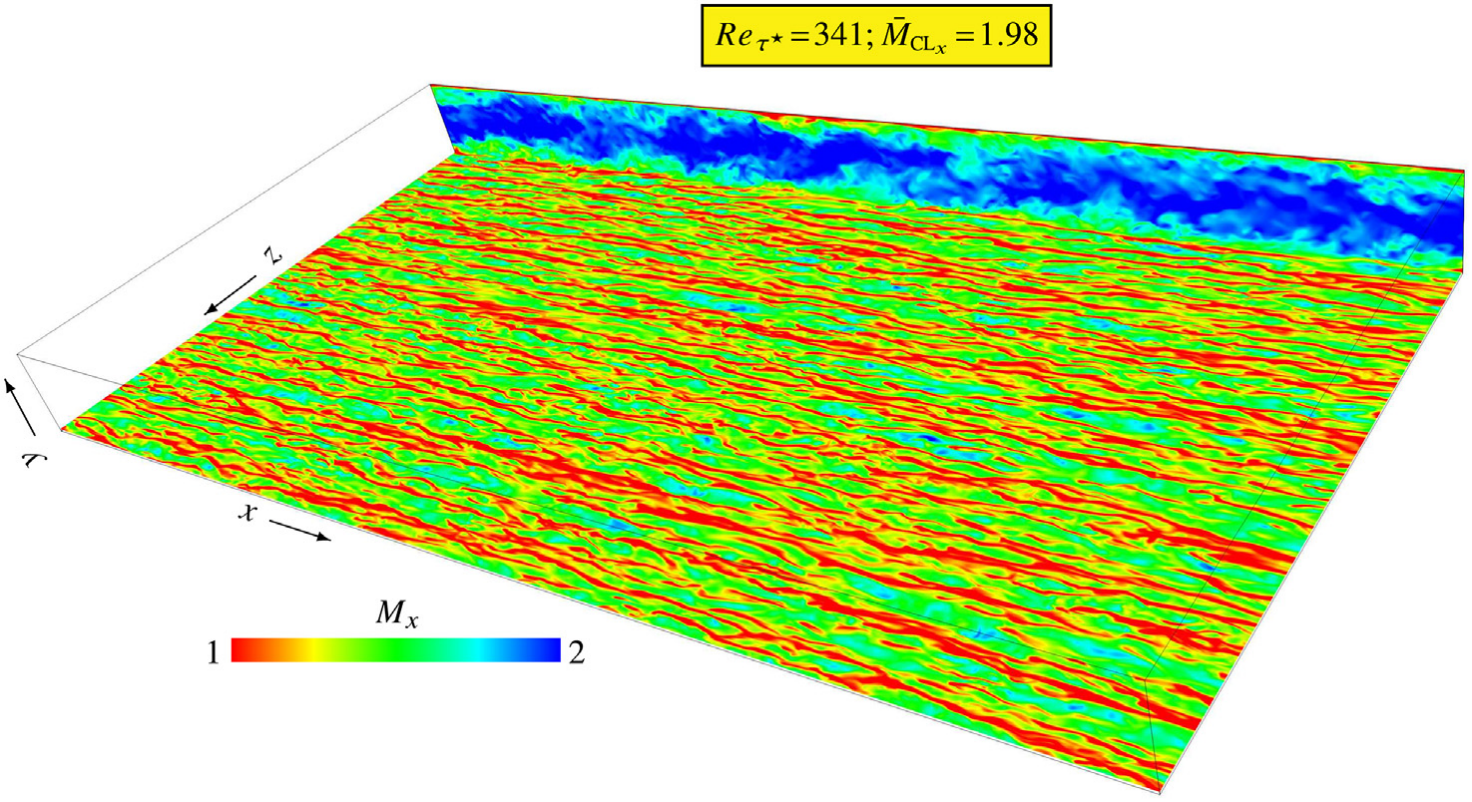
Computational domain and instantaneous streamwise Mach number $M_{x}$ levels (colormap limited in the range [1,2]) for the $\left(Re_{\tau^{★}},{\bar{M}}_{{\text{CL}}_{x}}\right)\;=\;\left(341,1.98\right)$ dataset (xz-plane at $y^{★}\;=\;19.76$ where the instantaneous snapshot levels are $0.44\;⪅\;M_{x}\;⪅\;2.09$ and xy-plane at the spanwise periodic boundary where the instantaneous snapshot levels are $0\;\leq \;M_{x}\;⪅\;2.31$).

**Fig. 4:**
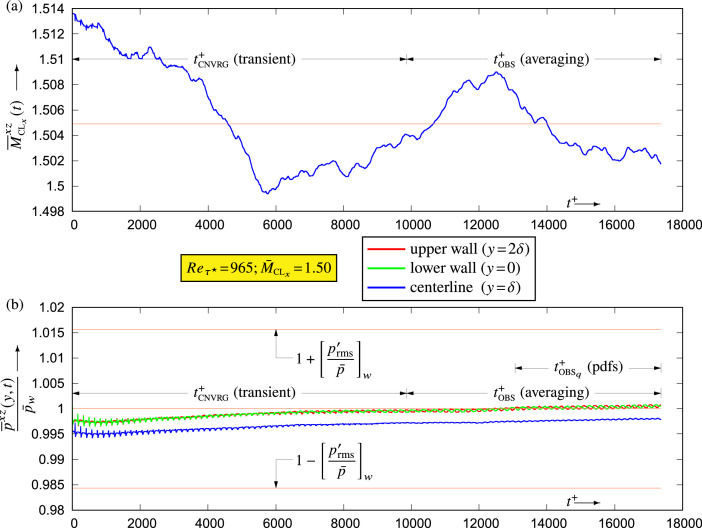
Typical time evolution of instantaneous ${\bar{\left(\cdot \right)}}^{xz}$-averages (in the homogeneous streamwise x and spanwise z directions) (a) of the centerline streamwise Mach number ${\bar{M}}_{{\text{CL}}_{x}}^{xz}\left(t\right)$ and (b) of static pressure ${\bar{p}}^{xz}\left(y,t\right)/{\bar{p}}_{w}$ at the walls and at centerline, for the $\left(Re_{\tau^{★}},{\bar{M}}_{{\text{CL}}_{x}}\right)\;=\;\left(965,1.50\right)$ dataset DNS computations, illustrating the elimination of the transient ($t_{\text{CNVRRG}}$) and the subsequent acquisition of statistics ($t_{\text{OBS}}$ and $t_{{\text{OBS}}_{q}}$) time-intervals (Table 1).

Direct numerical simulation (DNS) (§4.1) was used to compute the flow (Fig. 3) for each $\left(Re_{\tau^{★}},{\bar{M}}_{{\text{CL}}_{x}}\right)$-condition (Table 1). After calculating the flow for an initial time-interval $t_{\text{CNVRG}}$, sufficiently long to eliminate the transient (Fig. 4), the computations were continued for an additional time-interval $t_{\text{OBS}}$ with activated on-board sampling (§4.2) of dynamic and thermodynamic flow-variables for the acquisition of averages (moments) and of pdfs. Fully automated post-processing was then applied to these data, to calculate correlations and moments of the fluctuating field and standardized pdfs, and to computer-generate the datasets included in the present database (§4.3). The entire procedure was scripted to avoid human errors.

### 4.1 Solver and convergence

Following standard practice in the field [14], [17], [18], [20], [21], introduced by Coleman et al. [3], the flow was made streamwise invariant by the introduction of a body-acceleration $f_{{\text{V}}_{x}}\left(t\right)$ (space invariant but time-dependent) replacing the streamwise pressure-gradient necessary to counter skin-friction (budgets of numerous second-moment equations [6] and the Reynolds-stresses budgets in the subdirectories 1_PBs_profiles_and_budgets of the present datasets demonstrate negligible influence of the time-variation of $f_{{\text{V}}_{x}}$). The computational box (Fig. 3) is delimited by the lower (y=0) and upper (y=2δ) walls and the streamwise (x) and spanwise (z) periodic boundaries.

The DNS computations were run using a very-high-order accurate DNS solver [5], integrating the compressible Navier-Stokes equations with air thermodynamics and transport coefficients [6, p. 706]. The solver is massively parallel using hybrid MPI/OpenMP programming with verified scalabilty (up to 64736 cores) on several supercomputers [2], [11], [13], [19]. The computation and on-board statistics acquisition of the 25 flow-conditions included in the database required $∼\;250\times 10^{6}\;\text{corehours}$. Results with this solver have been carefully verified both by consistency checks [9] and by comparison with data by other authors [5], [8].

Define the bulk-averaging operator and the average bulk Mach and Reynolds numbers [3], [10]

(2) $$\begin{array}{c} {\left(\cdot \right)}_{\text{B}}:=\frac{1}{L_{x}L_{y}L_{z}}\underset{V}{\;\int \int \int \;}\left(\cdot \right)\;dv\;;\;M_{{\text{B}}_{w}}:=\frac{{\left(\bar{\rho u}\right)}_{\text{B}}}{{\bar{\rho }}_{B}\;{\bar{a}}_{w}}\;;\;Re_{{\text{B}}_{w}}:=\frac{{\left(\bar{\rho u}\right)}_{\text{B}}\delta }{{\bar{\mu }}_{w}} \end{array}$$

where V is the volume of the computational box with dimensions $L_{x}\;\times \;L_{y}\;\times \;L_{z}$ in the corresponding space directions (Table 1). The numerical procedure described in [5, (47,48), p. 791] fixes target $\left(Re_{{\text{B}}_{w}},M_{{\text{B}}_{w}}\right)$-conditions. These target values were carefully chosen during the construction of the database to built a $\left(Re_{\tau^{★}},{\bar{M}}_{{\text{CL}}_{x}}\right)$-matrix instead (data for the relation between $\left(Re_{\tau^{★}},{\bar{M}}_{{\text{CL}}_{x}}\right)$ and $\left(Re_{{\text{B}}_{w}},M_{{\text{B}}_{w}}\right)$ were interpolated/extrapolated from previously calculated datasets, learning as more datasets were made available).

### 4.2 Statistics acquisition and observation times

Semi-analytical velocity and temperature profiles were used to determine initial conditions for each new $\left(Re_{\tau^{★}},{\bar{M}}_{{\text{CL}}_{x}}\right)$-configuration [5, p. 790]. Instead of applying random perturbations of the velocity field [5, p. 790], the spatial organization of $\{\rho ,u_{i},T$} was interpolated from an existing simulation (at the nearest available conditions), with appropriate rescaling to fit the new target meanflow conditions. The initial part of the calculation ($t_{\text{CNVRG}}$) served to eliminate the transient perturbation associated with the approximate initialization (Fig. 4). After this initial transient, on-board sampling was activated and computations were continued to acquire converged statistics for an observation time-interval $t_{\text{OBS}}$ (Fig. 4).

A large number for moments ${\bar{q_{1}\cdots q_{n}}}^{txz}\left(t_{\text{OBS}},y\right)$ were acquired at each wall-normal station by a moving-averages approach

(3) $$\begin{array}{ccc} {\bar{q_{1}\cdots q_{n}}}^{txz}\left(t_{\text{OBS}}^{\text{NEW}},y\right) & = & \left(1-\frac{\Delta t_{s}}{t_{\text{OBS}}^{\text{NEW}}}\right){\bar{q_{1}\cdots q_{n}}}^{txz}\left(t_{\text{OBS}}^{\text{OLD}},y\right)+\frac{\Delta t_{s}}{t_{\text{OBS}}^{\text{NEW}}}{\bar{q_{1}\cdots q_{n}}}^{xz}\left(t_{\text{OBS}}^{\text{NEW}},y\right)\;;\\ t_{\text{OBS}}^{\text{NEW}} & = & t_{\text{OBS}}^{\text{OLD}}+\Delta t_{s} \end{array}$$

where $\Delta t_{s}$ is the sampling time-step (in the present datasets sampling was performed at each iteration, *ie* $\Delta t_{s}=\Delta t$), ${\bar{\left(\cdot \right)}}^{xz}$ denotes averaging in the homogeneous xz-directions, ${\bar{\left(\cdot \right)}}^{txz}$ denotes averaging in the homogeneous txz-directions, and generic q-notation is used for flow quantities.

Simultaneously, extreme events were identified for a large number of dynamic and thermodynamic quantities. Using again generic q-notation for flow quantities

(4a) $$\begin{array}{cc} & \;\;\;\;\;\;\;\;\;\;\;\;\;\;\text{symmetric}\;\bar{q}\left(y\right)=\bar{q}\left(2\delta -y\right)\;:\\ & q_{{\text{XTRM}}_{\min}}\left(t_{\text{OBS}}^{\text{NEW}},y\right)=\min\left(q_{{\text{XTRM}}_{\min}}\left(t_{\text{OBS}}^{\text{OLD}},y\right),\min_{xz}q\left(x,y,z,t_{\text{OBS}}^{\text{NEW}}\right),\min_{xz}q\left(x,2\delta -y,z,t_{\text{OBS}}^{\text{NEW}}\right)\right) \end{array}$$

(4b) $$\begin{array}{cc} & q_{{\text{XTRM}}_{\max}}\left(t_{\text{OBS}}^{\text{NEW}},y\right)=\max\left(q_{{\text{XTRM}}_{\max}}\left(t_{\text{OBS}}^{\text{OLD}},y\right),\max_{xz}q\left(x,y,z,t_{\text{OBS}}^{\text{NEW}}\right),\max_{xz}q\left(x,2\delta -y,z,t_{\text{OBS}}^{\text{NEW}}\right)\right) \end{array}$$

(4c) $$\begin{array}{cc} & \;\;\;\;\;\;\;\;\;\;\;\;\;\;\text{antisymmetric}\;\bar{q}\left(y\right)=-\bar{q}\left(2\delta -y\right)\;:\\ & q_{{\text{XTRM}}_{\min}}\left(t_{\text{OBS}}^{\text{NEW}},y\right)=\min\left(q_{{\text{XTRM}}_{\min}}\left(t_{\text{OBS}}^{\text{OLD}},y\right),\min_{xz}q\left(x,y,z,t_{\text{OBS}}^{\text{NEW}}\right),-\max_{xz}q\left(x,2\delta -y,z,t_{\text{OBS}}^{\text{NEW}}\right)\right) \end{array}$$

(4d) $$\begin{array}{cc} & q_{{\text{XTRM}}_{\max}}\left(t_{\text{OBS}}^{\text{NEW}},y\right)=\max\left(q_{{\text{XTRM}}_{\max}}\left(t_{\text{OBS}}^{\text{OLD}},y\right),\max_{xz}q\left(x,y,z,t_{\text{OBS}}^{\text{NEW}}\right),-\min_{xz}q\left(x,2\delta -y,z,t_{\text{OBS}}^{\text{NEW}}\right)\right) \end{array}$$

The sampling of extreme events allows the determination of the range covered by the pdf-bins, for acquisition of pdfs, which starts after an initial observation time (Fig. 4), required for a reliable evaluation of extreme events. The upper/lower halfchannel symmetrization in (4) is necessary to ensure symmetry of the lower/upper pdf-bins.

Regarding single-variable pdfs (subdirectories 2_pdfsq_single_variable_pdfs) $N_{\mathrm{bins}}$ of width Δq(y) were sampled in the range (fixed at the initial instant of pdfs sampling $t=t_{0_{{\text{OBS}}_{q}}}$)

(5a) $$\begin{array}{cc} q_{\min}\left(y\right) & :=q_{{\text{XTRM}}_{\min}}\left(y\right)-\frac{1}{10}\left(q_{{\text{XTRM}}_{\max}}\left(y\right)-q_{{\text{XTRM}}_{\min}}\left(y\right)\right) \end{array}$$

(5b) $$\begin{array}{cc} q_{\max}\left(y\right) & :=q_{{\text{XTRM}}_{\max}}\left(y\right)+\frac{1}{10}(q_{{\text{XTRM}}_{\max}}\left(y\right)-q_{{\text{XTRM}}_{\min}}\left(y\right)) \end{array}$$

(5c) $$\begin{array}{cc} \Delta q(y) & :=\frac{q_{\max}\left(y\right)-q_{\min}\left(y\right)}{N_{\mathrm{bins}}} \end{array}$$

(5d) $$Q_{\mathrm{bin}}\left(y,n\right):=\left[q_{\min}\left(y\right)+\left(n-1\right)\Delta q\left(y\right)-\frac{1}{2}\Delta q\left(y\right),q_{\min}\left(y\right)+\left(n-1\right)\Delta q\left(y\right)+\frac{1}{2}\Delta q\left(y\right)\right]$$

with the bin $Q_{\mathrm{bin}}\left(y,n\right)$ centered aroung $q_{\min}\left(y\right)\;+\;\left(n-1\right)\Delta q\left(y\right)$. Hits (occurences) $N_{\text{hits}}\left(n,y\right)$ are cumulatively recorded in each bin $Q_{\mathrm{bin}}\left(y,n\right)$. Notice the 20%-increase of the sampling-range ((5a), (5b)) to ensure that almost all future events remain inside the sampled bins (2 additional counters for hits outside of the range were included and verified that the associated probability is indeed 0 or negligibly small).

Joint 2-variable pdfs (subdirectory 3_pdfs2q_two_variable_joint_pdfs) were sampled on a restricted range $\left[{\bar{q}}_{1}-5q_{1\mathrm{rms}}^{\prime },{\bar{q}}_{1}+5q_{1\mathrm{rms}}^{\prime }\right]\times \left[{\bar{q}}_{2}-5q_{2\mathrm{rms}}^{\prime },{\bar{q}}_{2}+5q_{2\mathrm{rms}}^{\prime }\right]$, to reduce memory requirements. This range allows for an accurate description of the pdfs and for the calculation of the covariance, but not for higher-order correlations which are obtained directly by averaging (3).

### 4.3 Post-processing and datasets construction

At the end of the computations, all statistical data (§4.2) are assembled from the MPI-processes in a single data-structure, and upper/lower halfchannel symmetrization or antisymmetrization is performed, both for averages and pdf-bins. Standard Reynolds- and Favre-averaging relations are applied on moments (*eg* $\bar{q_{1}\cdots q_{n}}$ or $\bar{\rho q_{1}\cdots q_{n}}$) to obtain turbulent correlations (*eg* $\bar{q_{1}^{\prime }\cdots q_{n}^{\prime }}$ or $\bar{\rho q_{1}^{\prime \prime }\cdots q_{n}^{\prime \prime }}$). Similarly, hit-data in the pdf-bins are readily transformed to pdfs by normalization of the integral to 1, $\bar{q}$ and $q_{\mathrm{rms}}^{\prime }$ are computed by integration, and used to obtain the standardized pdfs which are included in the datasets. This procedure is performed by a post-processing module, which outputs automatically all the datafiles (comments and data) included in the present database.

## Limitations

The thermodynamic model for the present data uses, in line with most of the published work in the field [14], [17], [18], [20], [21] $c_{p}\left(T\right)\;=\;\text{const}$ specific heat-capacity (full description of thermodynamics and transport coefficients in [6, p. 706]).

## Ethics Statements

The authors have read and follow the ethical requirements for publication in Data in Brief. The current work does not involve human subjects, animal experiments, or any data collected from social media platforms.

## CRediT authorship contribution statement

**G.A. Gerolymos:** Conceptualization, Methodology, Software, Validation, Formal analysis, Investigation, Writing – review & editing, Visualization, Project administration, Funding acquisition. **I. Vallet:** Conceptualization, Methodology, Software, Validation, Formal analysis, Investigation, Writing – review & editing, Visualization, Project administration, Funding acquisition.

## Data Availability

Compressible turbulent plane channel DNS database
